# Composition in Sozodont, Etc.

**Published:** 1877-12

**Authors:** 


*Composition of Sozodont, Etc.*—In Germany, E. Hahn has published in book form the ingredients of over eleven hundred patent medicine preparations; the analyses of which were made by Drs. Hager, Wittstein, Rose, Chandler and others. According to these tests the reddish liquid of Sozodont contains a solution of 5 grammes of oil soap in 6 grammes of glycerine, 30 grammes of spirits, 20 grammes of water, perfumed with a few drops of oil of peppermint, oil of cloves, oil of cinnamon, and oil of anise, and colored with cochineal. The powder of Sozodont is a mixture of corbonate of lime, magnesia, and orris root.

Horn's Liton, which professes to be an infallible cure for toothache, contains: 5 parts of phosphate of lithia dissolved in 400 parts of alcohol.

---

### NOTES FROM PRACTICE.

